# Resource list for cognitive motor and sensory supports in persons with autism

**DOI:** 10.3389/fnint.2013.00007

**Published:** 2013-02-26

**Authors:** Kathleen A. Berger

**Affiliations:** ^1^Therapy Intensive Programs, Inc.Tallahassee, FL, USA; ^2^Rehabilitation Science Doctoral Program, College of Public Health and Health Professions, University of FloridaGainesville, FL, USA

## Introduction

This special issue, “Autism: the movement perspective,” includes several articles addressing cognitive motor differences in persons with autism. But, what does a person, or parent of a person, with autism do with this information? While numerous therapy organizations exist that address cognitive or motor issues separately, few organizations have combined cognitive and motor perspectives to uncover the hidden potentials of persons with autism. This commentary compiles a brief description of, and contact information for, a handful of therapeutic and/or educational organizations that address cognitive motor challenges, as well as sensory processing differences, in persons with autism. It is not an exhaustive list: I built it initially contacting organizations with which I am familiar and asked them to provide information, and recommend other similar organizations.

## Bodyspeaks

If you know someone who struggles to communicate or whose speech is limited, BodySpeaks may be able to help. I work with families, professionals, school districts and agencies and most importantly, those individuals who struggle to make their needs known. My approach to communication solutions is based on several important premises: (1) everyone communicates. It is important to set a vision for full communication; (2) communication partners play a vital role in the success of any alternative communication system, and (3) everyone uses multiple means to communicate.

I can help identify how a person communicates and interpret the meaning of their behavior. I can also help build their communication toward a system that offers the possibility of complex expression. I work at this in a variety of ways that you can see as you peruse the website. For those who have an established communication system, I work to strengthen their independence and reliability as a communicator.

For additional information: e-mail: marilynachadwick@gmail.com. Phone: 315-247-6772.

## HALO

Helping Autism through Learning and Outreach (HALO) is a 501 (c)(3) Non-profit organization located in Austin, TX supported by parents and professionals worldwide who are dedicated to the use of Soma® Rapid Prompting Method for persons with autism and similar disorders.

RPM is used to teach academics and communication is also taught in the process. The aim is to bring the student to maximum learning through the open learning channel and elicit the best out of the child to enable maximum output in that given time. As a student's cognitive and motor proficiency increases, the sophistication of a student's response improves. www.halo-soma.org.

## ICI

The Institute on Communication and Inclusion (ICI) at Syracuse University is a national and international leader in communication training and research for people with disabilities who do not demonstrate reliable verbal speech. Research, training and public information dissemination efforts focus on school and community inclusion, narratives of disability and ability, developing more effective and independent communication, and disability rights. Our initiatives stress the important relationship of communication to inclusion and our mission is based on the principle that “Not being able to speak is not the same as not having anything to say.” Contact the ICI at fcstaff@syr.edu, or visit our website http://ici.syr.edu.

## NMTSA

Neurologic Music Therapy Services of Arizona (NMTSA) is a non-profit organization in the Phoenix area that serves individuals with acquired or developmental Neurologic disorders, their families and support teams. NMTSA approaches autism as a psychomotor regulation sensory processing disorder. NMTSA provides Neurologic Music Therapy for children and adults as well as provides consultations and trainings in the community. NMTSA also has a school for children with autism—Assuming Competence Today (ACT School). For information about clinical services contact Executive Director Suzanne Oliver MT-BC, NMT at soliver@nmtsa.org or Clinical Development Specialist Melissa Lloyd MT-BC, NMT at mlloyd@nmtsa.org. For more information about ACT contact ACT Site Coordinator Bethany Jones MT-BC, NMT at bjones@nmtsa.org.

## TIP

Therapy Intensive Programs (TIP)/Kris' Camp, Inc is a therapy intensive, respite program for children with autism and their families. TIP's therapeutic philosophy approaches autism as a psychomotor regulation sensory processing disorder. Programs are provided for children as well as adults throughout the year. Additionally, Kris' Camp provides continuing education and training opportunities for educators, therapists, staff and families. For further information or to contact: www.kriscamp.org. Assistant Director: Leidy van Ispelen: Phone: (Mountain Time Zone): (801) 733-0721. Program Director: Michelle Hardy, MT-BC, NMT: Phone: (California) (619) 770-9314.

## WAPADH

Whittier Area Parents' Association for the Developmentally Handicapped (WAPADH), is a non profit, and non public agency, in Los Angeles County, CA. We provide speech and language, and augmentative communication services to both children and adults in California. We specialize in working with individuals with severe communication impairments that are also impacted by sensory and emotional needs. We incorporate strategies that support the use of both low and high technology needs, and motor planning. Our services are provided in the clinic and in the school setting, as well as through video chat. At WAPADH we connect with the individual's team to create a productive and communicative life style. WAPADH also provides trainings in the area of Communication, Communication Partner Skills, Facilitated Communication Training, and iPad use for communication. Our team consists of Speech and Language, Assistive Technology, and Communication Partners. For additional information please contact Darlene Hanson, Director of Communication Services, at 562-946-0467 xt, 105 or dghanson@me.com.

## Summary

While there are a large number of excellent therapy supports available to persons with autism, most of them focus on applied behavior and typical development theory, treating motor, and cognitive challenges as separate issues. Alternatively, the above organizations have a core philosophy addressing cognitive motor challenges as a unitary concept in persons with autism. I encourage persons with autism, and the parents of such persons, to explore the therapeutic opportunities offered by these, and what I expect will be a growing number of similar, organizations.

